# Competition with wind-pollinated plant species alters floral traits of insect-pollinated plant species

**DOI:** 10.1038/srep13345

**Published:** 2015-09-03

**Authors:** Floriane Flacher, Xavier Raynaud, Amandine Hansart, Eric Motard, Isabelle Dajoz

**Affiliations:** 1 CNRS, Sorbonne Universités, UPMC Univ Paris 6, INRA IRD, Univ Paris Diderot Paris 7, UPEC, Institute of Ecology and Environmental Sciences - Paris, UMR 7618, 7 Quai St Bernard, F-75005 Paris France; 2 Sorbonne Universités, UPMC Univ Paris 6, CNRS, INRA IRD, Univ Paris Diderot Paris 7, UPEC, Institute of Ecology and Environmental Sciences - Paris, UMR 7618, 7 Quai St Bernard, F-75005 Paris France; 3 CNRS, UMS 3194 CEREEP-Ecotron Ile de France, F-77140 Saint-Pierre-lès-Nemours; 4 Univ Paris Diderot Paris 7, Sorbonne Universités, UPMC Univ Paris 6, CNRS, INRA IRD, UPEC, Institute of Ecology and Environmental Sciences - Paris, UMR 7618, 7 Quai St Bernard, F-75005 Paris France

## Abstract

Plant traits related to attractiveness to pollinators (e.g. flowers and nectar) can be sensitive to abiotic or biotic conditions. Soil nutrient availability, as well as interactions among insect-pollinated plants species, can induce changes in flower and nectar production. However, further investigations are needed to determine the impact of interactions between insect-pollinated species and abiotically pollinated species on such floral traits, especially floral rewards. We carried out a pot experiment in which three insect-pollinated plant species were grown in binary mixtures with four wind-pollinated plant species, differing in their competitive ability. Along the flowering period, we measured floral traits of the insect-pollinated species involved in attractiveness to pollinators (*i.e*. floral display size, flower size, daily and total 1) flower production, 2) nectar volume, 3) amount of sucrose allocated to nectar). Final plant biomass was measured to quantify competitive interactions. For two out of three insect-pollinated species, we found that the presence of a wind-pollinated species can negatively impact floral traits involved in attractiveness to pollinators. This effect was stronger with wind-pollinated species that induced stronger competitive interactions. These results stress the importance of studying the whole plant community (and not just the insect-pollinated plant community) when working on plant-pollinator interactions.

A wide array of plant traits are sensitive to environmental conditions, either abiotic and biotic factors and their interplay. Modifications of abiotic resources can induce positive to negative effects on plant vegetative (e.g. plant biomass) and reproductive traits (e.g. flowers, fruits or seeds). For instance, some studies showed that the addition of soil nutrients (e.g. nitrogen or phosphorous) can lead to an increase in plant growth rates or biomass^1^,^2^,^3^,^4^, as well as flower^3^,^5^,^6^,^7^,^8^, fruit^6^,^8^,^9^, pollen^9^ or seed traits^6^,^7^,^8^. On the other hand some studies showed that a high level of nitrogen can lead to a decrease in root biomass^4^ while increasing plant growth and flower production^7^. Likewise, litter and compost addition or irrigation can induce intricate plant responses. For instance, litter inputs can induce negative to positive effects on biomass along years^10^ as well as species-specific response of floral traits^11^ just like water addition^12^. The effect of resources modifications can also be delayed according to plant life cycles^3^. These studies suggest that plant response to resources availability can be plastic (the change in phenotype being proportional to changes in environmental conditions) or adaptive with strategies mainly linked to plants’ life cycles. Within plant communities, interactions among plant individuals, especially competition between roots systems for water and nutrient acquisition, can lead to changes in the availability of such resources^13^ and to changes in allocation to vegetative or reproductive plant traits^11^,^14^,^15^,^16^. Therefore, the composition of plant communities can have strong effects on individual plant traits through competitive interactions for resources.

Variations in plant reproductive traits are especially important in animal-pollinated species because they condition plant-pollinator interactions^17^,^18^. Indeed, in insect-pollinated plants, pollinators are attracted to flowers and associated rewards. On the one hand, flowers, varying in their number, size, color or smell, offer an advertising display that induces visits of various pollinators^17^,^19^. On the other hand, floral rewards (e.g. nectar, pollen) tend to favor the repetition of visits as they are major components of pollinators’ diet by supplying proteins, sugars and amino-acids^17^. The quantity and quality of such floral traits involved in pollinator attraction can have strong impacts on pollinator behavior. Indeed, several studies have shown that plant species exhibiting a greater floral display size, (i.e. the total number of opened flowers at a time) or producing numerous, large flowers and/or greater rewards (in quantity or quality) are more visited by pollinators than other present plant species^20^,^21^,^22^,^23^,^24^,^25^. Various experiments showed that a greater pollinator attractiveness, subsequently to increased floral traits through resource addition, can enhance pollinator visitation^6^,^7^,^8^,^26^ that may lead to better reproductive success^6^,^8^. However, to date, most studies linking changes in soil resources to floral traits and pollinator response considered the impact of nutrient addition while interactions between plants, especially belowground competition, could also be of great importance. In an experiment looking at different floral traits involved in attractiveness, Baude *et al.*^11^ set up binary mixtures of insect-pollinated plant species and found that floral traits of one focal species depended on the other species present in the mixtures. More precisely, the total nectar sugar content of a focal species decreased when growing in presence of a stronger competitor. Therefore, interactions between insect-pollinated species could influence floral traits involved in pollinator attraction.

Natural plant communities always comprise species with a variety of pollination modes such as animal-pollinated and abiotically pollinated plants, although the latter are almost never taken into account in studies on plant-pollinator networks. However, the flower production of a particular insect-pollinated species can be negatively impacted by competitive interactions induced by a wind-pollinated plant competitor^15^,^27^. To our knowledge, the consequences of interactions between insect- and wind-pollinated species on floral traits involved in attractiveness to pollinators still need investigations, especially with more focus on floral rewards.

The objectives of our study were to assess how allocation to several floral traits involved in attractiveness to pollinators (e.g. flower production, flower size and floral rewards) could be affected by the presence of different wind-pollinated species. Especially we wanted to investigate the effect of different intensities of competitive interactions. To do so, we set up a pot experiment in which we grew three insect-pollinated plant species (*Echium plantagineum*, *Lamium purpureum* and *Lotus corniculatus*) in binary mixtures with four wind-pollinated species (*Agrostis capillaris, Chenopodium album, Holcus lanatus* and *Plantago lanceolata*) so that insect-pollinated species were submitted to a panel of belowground interactions for abiotic resources.

Our hypothesis were that (1) the presence of wind-pollinated competitors should have negative impacts on floral traits of insect-pollinated species and (2) the magnitude of this effect should differ according to different competition intensities induced by the presence of wind-pollinated species.

## Results

### Intensity of competitive interactions

Mean log response ratios (ln RR^28^), estimators of competition intensity, are summed up in Table 1. As indicated by ln RR values, the three insect-pollinated focals were submitted to various intensities of competition. *E. plantagineum* and *L. purpureum* followed the same pattern of response. For both species and whatever the biomass measurement (aboveground, belowground or total biomass), ln RR values (as well as focals’ biomass) were significantly higher in presence of *C. album*, in opposition to mixtures with *H. lanatus* for which ln RR had the lowest values (P ≤ 0.024 for both species, Table 1, Supplementary Fig. S1 for biomass). Mixtures with *C. album* even seemed to provide better growth conditions than monocultures of the two focal species with positive values of ln RR (Table 1). For intermediate levels of competition intensity, ln RR values suggest a global pattern where intensity of competition is stronger in mixtures with *A. capillaris* than with *C. album* but weaker than with *P. lanceolata,* the second strongest competitor for *L. purpureum and E. plantagineum* (except for ln RR values calculated with belowground biomass of *L. purpureum,* Table 1). According to these results, *L. purpureum* and *E. plantagineum* experienced the following panel of growing intensity of competition:


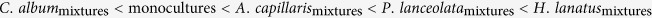


The response of *L. corniculatus* didn’t follow the same pattern. Monocultures showed the highest ln RR values among all treatments (Table 1). In consequence, all wind-pollinated species induced negative competitive interactions for this focal species ranging from weak effects in mixture with *C. album* to strong effects in mixture with *P. lanceolata* or *A. capillaris* (Table 1).

As we are mainly interested in the effect of competition intensity induced by wind-pollinated species rather than on competitor identity, the log response ratio (ln RR) has been used as an explanatory variable in the following results. However, because ln RR values were obtained from biomass measurements at the end of the experiment, we used ln RR (calculated from total biomass, see in Methods) as an explanatory variable only for total floral traits (i.e. summed at the end of the experiment, see Flower traits, Nectar traits and Data analysis sections in Methods). We kept the competitor identity as an explanatory variable for daily floral traits see Flower traits, Nectar traits and Data analysis sections in Methods). Therefore, in the following results, ‘competition intensity’ will equally refer to 1) ln RR values, 2) the presence of a particular competitor in the mixture with the focal species. An increase in competition intensity can thus mean a decrease in ln RR values or the presence of stronger competitors. For the sake of clarity, the results for daily floral traits will be ordered along the above panel for the three focal species, even if the pattern of response was different for *L. corniculatus.*

### Flower traits

**Figure 1:**
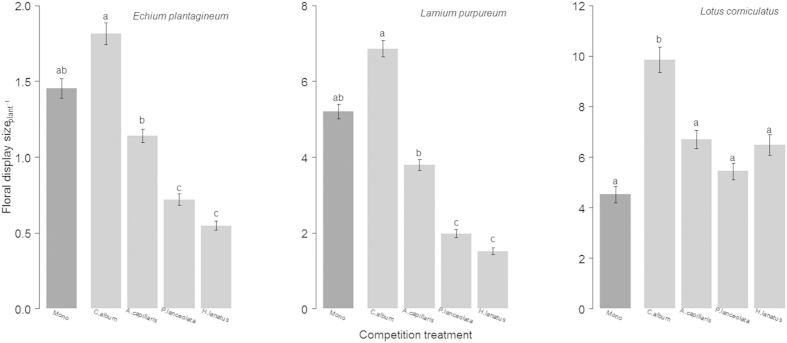
Mean (+/−standard error) floral display size per plant of *E. plantagineum, L. purpureum,* and *L. corniculatus* in mixture with the competitors. “Mono” refers to monocultures of the focal species. Wind-pollinated species are ordered according to increasing intensity of competitive interactions (see Results). Different letters indicate significant differences (i.e P ≤ 0.006 for *E. plantagineum*, P ≤ 0.001 for *L. purpureum* and P ≤ 0.040 for *L. corniculatus*) after pairwise comparisons (Tukey^57^) and adjustment of p-values (Holm method^56^).

At the end of the experiment, a total of 807, 2053 and 1075 flowers were sampled for *E. plantagineum, L. purpureum,* and *L. corniculatus,* respectively. For *E. plantagineum and L. purpureum* there was a significant effect of competition leading to a decrease of floral display size, daily flower production (not shown) and total flower production (total number of flower produced at the end of the flowering period, see Methods) when competition intensity increased. Indeed, floral display size and daily flower production tended to be greater in presence of *C. album* (even greater than in monocultures; although not significant) while the presence of stronger competitors, such as *H. lanatus*, induced a strong decrease in both traits (Fig. 1 for floral display size, the daily flower production followed the same pattern). Likewise, for both species, the total flower production decrease as ln RR values decrease, suggesting lower total flower production in condition of stronger competitive interactions (P < 0.001 Supplementary information Fig. S2). For *L. corniculatus*, there was also a significant effect of the competition treatment. Especially, floral display size and daily flower production were greater in presence of *C. album* (Fig. 1 for floral display size, the daily flower production followed the same pattern). However, both traits tended to be lower in monocultures (Fig. 1 for floral display size, the daily flower production followed the same pattern). On the other hand, the total flower production decreased according to ln RR values (P < 0.001, Supplementary information Fig. S2). For all three species, there were significant effects of the date (for the floral display size only, all P < 0.001) and of the interaction terms for both floral display size and daily flower production (all P < 0.001).

For all three focal species, flower size was affected by the competition treatment (*E. plantagineum:* F_4,772_ = 7.35, P < 0.001, *L purpureum*: F_1,1936_ = 8.96, P < 0.001; *L corniculatus:* F_4,1036_ = 4.69, P < 0.001, Supplementary information Fig. S3). Plants of *E. plantagineum* and *L. purpureum* produced smaller flowers as competition intensifies. For *L. corniculatus*, monocultures and mixtures with *C. album* produced the smallest flowers (see Supplementary information Fig. S3). For flower size, there was also a significant effect of the date for all three species (all P ≤ 0.008).

### Nectar traits

There was no significant effect of the competition treatment on the daily concentration of nectar per flower and the daily volume of nectar per flower for both *E. plantagineum* and *L. corniculatus* (P > 0.05). *L. purpureum,* however, was significantly affected by the presence of stronger competitors for both variables (F_4,1450_ = 2.53, P = 0.04 for the daily concentration and F_4,1648_ = 4.47, P = 0.001 for the daily nectar volume).

**Figure 2:**
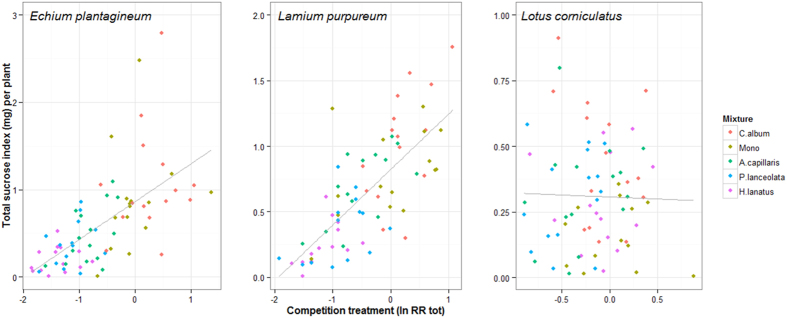
Linear regression between the total sucrose index (mg per plant) and mean ln RR values per plant calculated from total biomass (P < 0.001 with transformed data, for E. *plantagineum* (R^2^ = 0.44) and L. *purpureum* (R^2^ = 0.57) only). The grey line corresponds to the estimated model while dots represent the data. Ln RR values are associated to mixtures in the legend.

As the daily flower production was affected by the competition treatment, the daily sucrose index of nectar (taking into account all produced flowers per day and not only sampled flowers, see Methods) decreased in presence of strongest competitors for both *E. plantagineum* and *L. purpureum* (F_4,194_ = 13.36, P < 0.001and F_4,731_ = 5.52, P < 0.001 respectively). Therefore, the daily allocation of sucrose to nectar was lower when competition intensified. The same pattern was clearly observed for the total allocation of sucrose to nectar. Indeed, the total sucrose index at the end of the flowering period tended to decrease with ln RR values (*E. plantagineum* F_1,47_ = 57.04, P < 0.001, *L. purpureum*: F_1,48_ = 83.81, P < 0.001; Fig. 2, see Methods). We found the same pattern for daily (*E. plantagineum* F_4,199_ = 9.38, P < 0.001; *L. purpureum* F_4,800_ = 8.05, P < 0.001) and total volume index (F _1,46_ = 30,71, P < 0.001, F _1,48_ = 102,74 P < 0.001 respectively). For *L. corniculatus,* the daily sucrose index and the daily volume index were significantly affected by the competition treatment (F_4,255_ = 3.48, P = 0.009 and F_4,254_ = 3.47, P = 0.009 respectively) with lower values in monocultures. However both index of nectar were not affected by increasing competition intensity (both P > 0.05, see Fig. 2 for total sucrose index). There were significant effects of the date for the daily concentration of nectar (P ≤ 0.02 for *E. plantagineum* and *L. purpureum*) and the daily nectar volume (*L. purpureum* only P < 0.001). There was a significant effect of the interaction term (competition:date) for the daily nectar volume (for *E. plantagineum and L. corniculatus, all* P ≤ 0.01). For both daily index, there were significant effects of the date (for *L. purpureum* only, P < 0.001) and of the interaction term (competition:date) (for *E. plantagineum and L. corniculatus*, all P ≤ 0.002).

## Discussion

Literature data have mostly focused on the relations between plant attractiveness to pollinators and abiotic conditions and suggest that response of attractiveness traits is complex and species-specific as positive, neutral and negative effects have been reported for the effects of water or nutrient addition on flower productionp^3^,^7^,^11^,^12^. Because competitive interactions between plants can translate into changes in resources availability between competitors^13^,^29^, we studied their impact on floral traits of three insect-pollinated species. Moreover, we focused on wind-pollinated species as competitors in order to see how species that do not interact with pollinators for their reproduction could alter attractiveness traits of animal-pollinated focal species.

As expected, competition with wind-pollinated species affected plant biomasses and floral traits for all three investigated insect-pollinated species. Especially, we found that the stronger the competitive interaction was (i.e., high ln RR values), the stronger the impact on floral traits.

Differences in biomass allocation patterns, especially belowground, can suggest different competitive abilities in plants^29^. Indices of competition, such as the log response ratio (ln RR) are frequently used as they are good tools for summarizing and interpreting competitive interactions between plant species^28^. Here, the ln RR values obtained for each mixture indicate that the wind-pollinated competitors exposed the insect-pollinated focals to different competition intensities. These ln RR values also suggest that the two annual species of our experiment, *L. purpureum* and *E. plantagineum,* faced a similar panel of growing intensity of competition, with *C. album* being a weaker competitor, *A. capillaris* an average competitor and *P. lanceolata* and *H. lanatus* being stronger competitors. Measures of competitors’ biomass grown in mixtures with *L. purpureum* or *E. plantagineum* could explain these differences. Indeed, in these mixtures, plants of *H. lanatus* produced the greatest biomass among the four competitor species. Even though greater biomasses, especially greater root systems, are not always associated with greater competitive abilities^30^, larger root systems can be related to greater soil space occupation^31^ and/or greater resource uptake^29^ thus limiting access to resources for neighbouring plants. Likewise larger individual plants can induce stronger effects on target plants than smaller ones^32^. Here, *H. lanatus* is a strong competitor (so, with high competitive abilities) because its presence (its biomass) probably led to a strong limitation of the biomass production of the two focal species (lower ln RR values) through strong space occupation and/or greater nutrient depletion. In contrast, *C. album* individuals tended to have a positive effect on the biomass of these two focals species. Facilitative interactions between plants can be observed through modifications of soil components (e.g. moisture, nutrients)^33^ or enhancement of seedling establishment. However here, the positive effect of *C. album* may probably be due to low biomass production rather than facilitation. This may have favoured greater space/nutrient exploitation by the two focal species, and thus higher allocation to biomass than in the other treatments (including focals monocultures). So in this study, we consider that the competitive abilities of plant species are more a consequence of their biomass production (even if biomass production can also result from higher competitive abilities). In the case of *L. corniculatus,* response patterns were different. Mixture with *C. album* appart, *H. lanatus* behaved as an intermediate competitor in spite of its important biomass production (especially root biomass). Moreover, even though the range of ln RR values was narrower for this species (compared to *L. purpureum* for example), all wind-pollinated species had a small negative effect on *L. corniculatus,* compared to monocultures, and the strongest competitors were *P. lanceolata* and *A. capillaris*. However, biomass measurements indicate that only *L. corniculatus* belowground biomass was altered by the presence of a competitor and aboveground biomass was unaffected (see Supplementary information Fig. S1). Some characteristics of *L. corniculatus* could have mediated this different response to competition compared to the two other species. First, *L. corniculatus* is a legume species and, although we did not quantify them, *L. corniculatus* roots showed nodules, indicating that nitrogen fixation did occur in our experiment. *L. corniculatus* could thus have accessed to the atmospheric N pool^34^ so that it was only slightly affected by competition compared to the two other species. Furthermore*, L. corniculatus* has a perennial life cycle that can induce a different timing of response to competition as well as different allocation patterns compared to plants having annual life cycles^3^,^35^. Initially, the experimental design contained a second perennial plant, *Mimulus guttatus* DC. (synonym *Erythranthe guttata* (Fisch. ex DC.) G.L. Nesom; see Supplementary Table S1), that might have provided elements to disentangle the respective roles of perennial life cycle and nitrogen fixation in *L. corniculatus*. Unfortunately, only 15 out of 75 *Mimulus guttatus* plants flowered, and half of the flowers (25/49 total flowers) were produced by only two plants. As a consequence we decided not to include this species in this work.

Moreover, we should keep in mind that these conclusions rely on final harvests of biomass when some studies suggest regular harvest along experiments to better assess the dynamics of competitive interactions^36^.

Most of the floral traits measured in this experiment were affected by the competition treatment. Higher conditions of competition (lower ln RR values induced by the presence of *H. lanatus*) had the greatest impact on *E. plantagineum* and *L. purpureum,* by reducing flower and reward traits. To date, studies that have looked for links between attractiveness traits and environmental conditions have mostly focused on the effects of abiotic conditions and showed a sensitivity of attractiveness traits to nutrients and water availability^3^,^6^,^9^,^12^ or litter and compost additions to soil^11^,^26^. If modifications of abiotic conditions can alter species attractiveness to pollinators, it is not surprising that biotic interactions such as competition, that mediate abiotic resources availability, have similar effects^15^. Flower and nectar production can be relatively costly for a single plant^37^,^38^,^39^ so that allocation to reproductive structures might be modified in a context of competition with limited access to nutrients. Here, the lowest ln RR values, calculated from mixtures in presence of *H. lanatus,* suggest that this species may have reduced the availability of soil resources to *E. plantagineum* and *L. purpureum* and thus daily as well as total allocation of plants to floral traits. Overall, this could be responsible for lower resources allocation to reproductive traits. Conversely, *C. album*, led to higher flower and nectar production than in monocultures. A greater resources availability or space, due to the reduced biomass of the competitor might have led to better growing conditions for the insect-pollinated species, resulting in better resource acquisition (as confirmed by higher ln RR values) and increased allocation to reproductive structures.

In the case of *L. corniculatus*, while ln RR values suggest stronger competition (albeit limited) in presence of wind-pollinated species compared to monoculture, some floral traits were lowest in monoculture and in mixtures with *P. lanceolata* (which is the strongest competitor for *L. corniculatus* based on ln RR values) compared to the other mixtures. This suggests that, in contrast to the two other insect-pollinated species, allocation to reproduction was not related to biomass allocation. In Wurst & Van Beersum^40^, monocultures of *L. corniculatus* can have higher biomass and produce more flowers than in mixture with *H. lanatus,* which is not in accordance with the observed pattern here. Considering the cost of N_2_ fixation suggested in some studies (in term of C allocation to symbiont^41^), plants in monocultures in our experiment might have allocated less photosynthetates to floral traits leading to the observed decrease in monocultures. However, the study of floral traits per unit of biomass (total floral traits divided by the final biomass) revealed that *L. corniculatus* might have a more adaptive response to competition while the two other focal species might have a ‘purely’ plastic response to resource availability. Indeed, for *L. corniculatus,* floral traits per unit of biomass tend to be higher when competition intensity increases, showing a possible strategy to better attract pollinator in condition of competition. However, as we only have biomass data at the end of the experiment (and a final biomass can not only be considered as a sum of biomass like for total produced flowers, for instance), we believe that further investigations are needed to conclude on these effects.

Here we focused on flower and nectar production while other attractiveness traits could also be affected by competitive interactions. For example, plant pigments or volatile compounds involved in flower colours^42^ and scents, relative amounts of different sugars or amino-acids content in nectar^12^,^43^ and pollen quantity and or quality^9^, are all sensitive to resource variations, and could be affected by competitive interactions. Even though we observed a negative impact of competition on some floral traits involved in attractiveness to pollinators, the response of floral traits can be complex and species-specific^3^,^11^,^12^. Moreover, we interpret our results in a context of exploitative competition through soil resources depletion while other competitive mechanisms (e.g. interference through allelochemicals)^13^,^29^ could conjointly influence plant response. As ln RR values did not differ among total or belowground biomass and root competition is often stronger than shoot competition (especially with grass competitors^44^), our results are mainly interpreted in a context of belowground competition. However further investigations are needed to better assess the overall impact of plant competition (aboveground as well as belowground) on floral traits involved in attractiveness to pollinators.

Variations in attractiveness traits are known to strongly impact pollinator visitation patterns and on a larger scale pollination service. Indeed, greater plant attractiveness can enhance the frequency or number of flower visits: most pollinators are preferentially attracted to plants producing numerous, large flowers and/or greater rewards (in quality or quantity)^20^,^21^,^22^,^23^,^24^,^25^,^45^. Larger floral display size can also influence the abundance of visiting pollinators^46^. Likewise, the pattern of pollinator visits per plant can be correlated to the total nectar production per plant^47^. However, many flowering plants are pollen limited, therefore an increase in pollination intensity (e.g. a greater pollen deposit on stigmas) can enhance plant fecundity (i.e. greater fruit and/or seed set)^48^. As a consequence, our results suggest that a decrease in floral traits involved in pollinator attractiveness due to plant competition could have negative impacts on pollinator visits, reducing plant reproductive success. However further experiments are needed to test such hypothesis. Nevertheless, this study emphasises the importance of 1) taking into account species other than insect-pollinated ones in plant-pollinator network studies, and 2) linking above ground and below ground interactions to better understand plant-pollinator networks. This is in concordance with some research initiated on the impact of soil micro-organisms on pollinator visits through variations of floral traits^49^,^50^. Given our results, future research is needed on plant-soil or plant-plant interactions that may lead to modifications of floral traits involved in attractiveness to pollinators.

## Methods

Our objectives were to study how attractiveness traits of insect-pollinated plants are affected by the presence of neighbouring wind-pollinated plant species. To do so, we set up a greenhouse experiment in which we grew seven plant species in binary mixtures in pots.

### Plant species

Seven plant species (3 insect-pollinated plants and 4 wind-pollinated plants) with close ecological preferences (based on Ellenberg index for British Plants^51^) were selected: *Echium plantagineum* L., *Lamium purpureum* L., and *Lotus corniculatus* L., for insect-pollinated focals and *Agrostis capillaris L., Chenopodium album* L., *Holcus lanatus* L. and *Plantago lanceolata* L. for wind-pollinated competitors (for plant species description, see Supplementary Table S1).

### Experimental set-up

In March 2012, seedlings of all species were planted in plastic pots (14 cm Ø; 1.5L, Puteaux SA, France) in sandy soil (pH = 6). The soil was taken from a grassland site (CEREEP-Ecotron Ile-de-France, St Pierre-lès-Nemours, France) and was sieved (<4 mm) to remove rocks and plant material. Six plant individuals were placed in each pot to form two-species mixtures with three individuals of one insect-pollinated species in alternation with three individuals of one wind-pollinated species. We also set up control monocultures with six individuals of the same species (insect-pollinated or wind-pollinated). Each mixture was replicated 5 times, making a total of 95 pots (5 × 3 monocultures of insect-pollinated species, 5 × 4 monocultures of wind-pollinated species, 5 × 4 × 3 binary mixtures). Pots were randomly placed in a greenhouse (CEREEP-Ecotron Ile-de-France, St Pierre-lès-Nemours, France) and their position was changed each week. Plants were watered daily by sub-irrigation (flood floors, DIMAC SAS, France). Air temperature in the greenhouse followed outdoor conditions but was maintained above 18 °C when low temperatures occurred. Photoperiod was initially set at 12-hours per day through natural light and sodium lamps when necessary (i.e. when solar irradiation was under 200 watt/m^2^/hour; HS2000 Hortilux Schréder, The Netherlands). It was adjusted to 16-hours per day to allow for the blooming of *L. corniculatus*, a long-day flowering species. Because we were mainly interested by belowground competition in this study, we took special care to check that plant foliage did not overlap between individuals all along the experiment. When plant foliage did overlap (especially in mixtures with *L. corniculatus*) plant supports were put in to separate plant individuals and thus limit aboveground competition (i.e. for light).

### Floral traits of insect-pollinated species involved in attractiveness to pollinators

#### Flower traits

The flower traits measured every day were the number of newly opened flowers, plant’s floral display size and the size of the newly opened flowers. To assess the daily flower production (newly opened flowers per day and per plant), floral buds ready to opened were marked the evening before each sampling date, on all individuals of every insect-pollinated plant species. Buds actually opened on a sampling date were counted. Floral display size was calculated as the total number of opened flowers per day and per plant. Among the newly opened flowers (less than 14h-old), up to three flowers per plant were randomly selected to measure flower size and nectar traits (see below). Flower size measurement consisted in measuring corolla size (mm) with a digital caliper (Digit-Cal MK IV, Brown&Sharpe, USA) from the bottom to the tip of the corolla for *E. plantagineum* or to the tip of the keel for *L. corniculatus*. For *L. purpureum,* flower size was measured as the length of the corolla tube only (from the bottom of the corolla to the bottom of the upper lip of the corolla) in order to avoid errors due to strong variability in the opening angle of corolla’s upper lip (personal observations).

For all plants, the total number of produced flowers at the end of the flowering period was calculated by summing the daily flower production over the whole flowering period.

#### Nectar traits

For each plant, nectar volume and nectar sugar content were measured on up to three newly opened flowers, after flower size measurements (see above). This ensured that nectar traits were measured on flowers of the same age to limit variations due to flower age^52^. Nectar was sampled using microcapillary tubes (0.5 μL or 1 μL; Minicaps end to end, Hirschmann laborgeraete, Germany) and nectar volume was calculated by measuring the length of liquid in the microcapillary tube with a digital caliper (Digit-Cal MK IV, Brown&Sharpe, USA) (μL.flower^−1^.day^−1^). Daily sugar concentration was determined with hand-held refractometers (Eclipse 45–81 and Eclipse 45–82, Bellingham+Stanley Ltd., UK) calibrated using sucrose solutions (30% and 50% brix). Because nectar not only contains sucrose but also other sugars, our concentration measurements correspond to sucrose equivalent. However, for the sake of brevity, we will only use in the following the term sucrose in reference to “sucrose equivalent”. When nectar volumes were too small to be measured by the refractometer (<0.5 μL), samples were diluted in Milli-Q water before measurement. If concentration measurements could not be done right after sampling, microcapillary tubes were stored in a refrigerator at 4°C and measured within the next two hours. Because only up to three flowers per plant were sampled, we decided to calculate volume and sucrose indices taking into account the number of flowers produced per plant^47^. The daily volume index per plant was assessed by multiplying the average nectar volume per flower per day with the number of flower produced per plant per day. The daily amount of sucrose allocated to nectar per plant was estimated by multiplying the average concentration of sucrose in nectar per flower per day by the average volume of nectar per flower per day. This daily amount was multiplied by the number of flowers produced per plant per day, giving a daily sucrose index per plant. All daily floral traits (per flower or at the plant scale) can give information on the plant allocation to reproduction all along the flowering period. However, in order to have a global assessment of reward production and plant energy allocation, daily indices were summed along the whole flowering period as total indices^11^.

#### Plant traits

At the end of the flowering period of each insect-pollinated plant species (on the 3^rd^ of May 2012 for *L. purpureum* and on the 2^nd^ of June 2012 for *E. plantagineum* and *L. corniculatus*), above- and belowground biomass of all individuals was harvested. Concerning belowground biomass, we took care of separating root systems of each species. Plant biomasses were oven-dried (65°C, 48h) and weighted (g.plant^−1^).

#### Competitive interactions

In order to estimate the intensity of competitive interactions between each focal insect-pollinated plant and its wind-pollinated competitors we calculated the log response-ratio (ln RR) as an index of competition^28^. This index is defined as:


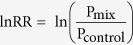


where P_mix_ is the biomass of a focal plant when grown in mixtures and P_control_ is the biomass of a focal plants in monoculture pots. In order to have a good assessment of the ln RR as well as a variance, ln RR values for each treatment were calculated as means of all possible combinations of each focal plant in a mixture divided by each focal plant in a monoculture. Because three focal plants were present in mixtures, we considered monocultures as ‘mixtures’ of 3 focal plants with 3 ‘competitor’ plants of the same species. Values of this index are symmetrical around zero with positive values indicating that focals grow better in mixture (i.e. focals are better competitors) and negative values indicating that focals’ growth is negatively affected by competitor (i.e. focals are lower competitors). Ln RR values were calculated from aboveground and belowground biomass but only ln RR calculated from total biomass were used to study the effect of competition on final floral traits as it is a better integrator of competition within both compartments.

#### Data analysis

All statistical analyses were performed using R 3.1^53^. Linear mixed-effects models were fitted to all measured traits (*nlme* R package^54^), with the exception of floral display size and total flower production that were fitted to generalized mixed-effect models with Poisson probability distribution and log link function (*lme4* R package^55^). As ln RR values are calculated from final biomass here, this may be relevant to study the response of total floral traits (values summed all along the flowering period for each plant to obtain a total value per plant) to competition but not for daily floral traits as competition can be dynamic along plant lifespan^36^. As a consequence models were fitted with two different explanatory variables: ln RR values calculated from total biomass as a fixed effect for total floral traits (i.e. total flower production, total sucrose index, total volume index) and wind-pollinated species identity as a fixed effect for daily floral traits (i.e. floral display size, flower size, daily sucrose concentration in nectar, daily nectar volume, daily volume index, daily amount of sucrose in nectar, daily sucrose index). The date was also set as a fixed effect for daily floral traits to take into account the effect of plant age. In all models, pots and date (for the repeated measures on plants) were set as random effects. For linear mixed models, data were transformed using log (e.g. floral traits involving nectar volume), square or square root (e.g. flower size, floral traits involving sucrose concentration) transformations, when necessary. Daily data were then analysed through analysis of covariance (ANCOVA). For total data, whose values were summed all along the flowering period for each plant to obtain a total value per plant, analyses of variance (ANOVA) were performed on these total values. When significant differences were detected, post-hoc comparisons were performed (Tukey all-pair comparisons, Holm method for p-value adjustment were used^56^, *multcomp* R package^57^). For the date effect or the interaction term, only significant effects are reported. Because generalized mixed-effect models (glmer, floral display size and total flower production) do not provide p-values, pairwise comparisons with Holm method for p-value adjustment were used^56^.

## Additional Information

**How to cite this article**: Flacher, F. *et al.* Competition with wind-pollinated plant species alters floral traits of insect-pollinated plant species. *Sci. Rep.* **5**, 13345; doi: 10.1038/srep13345 (2015).

## Supplementary Material

Supplementary Information

## Figures and Tables

**Table 1:** Mean ln RR values (+/−standard error) per treatment for *E. plantagineum*, *L. purpureum* and *L. corniculatus* in monocultures and mixtures with the wind-pollinated competitors. For each insect-pollinated focal, p-values indicate significant differences between treatments (ANOVA, *E. plantagineum* N = 1125, *L. purpureum* N = 1125, *L. corniculatus* N = 1125).

	ln RR					F value	p-value
	Monoculture	Mixture _*H. lanatus*_	Mixture _*P. lanceolata*_	Mixture _*A. capillaris*_	Mixture _*C. album*_		
*E. plantagineum*							
Aboveground biomass	0 (+/−0.01)	−1.16 (+/−0.01)	−0.86 (+/−0.01)	−0.60 (+/−0.01)	0.28 (+/−0.01)	F_4,20_ = 54.31	0.001
Belowground biomass	0 (+/−0.04)	−1.96 (+/−0.03)	−1.77 (+/−0.03)	−1.23 (+/−0.04)	0.39 (+/−0.04)	F_4,20_ = 20.73	0.001
Total biomass	0 (+/−0.02)	−1.43 (+/−0.02)	−1.15 (+/−0.02)	−0.82 (+/−0.02)	0.27 (+/−0.02)	F_4,20_ = 46.97	0.001
*L. purpureum*							
Aboveground biomass	0 (+/−0.02)	−1.00 (+/−0.02)	−0.81 (+/−0.02)	−0.22 (+/−0.02)	0.44 (+/−0.02)	F_4,20_ = 19.08	0.001
Belowground biomass	0 (+/−0.05)	−1.32 (+/−0.04)	−0.86 (+/−0.04)	−1.00 (+/−0.04)	−0.42 (+/−0.04)	F_4,20_=3.55	0.024
Total biomass	0 (+/−0.03)	−1.15 (+/−0.02)	−0.88 (+/−0.03)	−0.49 (+/−0.03)	0.19 (+/−0.02)	F_4,20_ = 12.74	0.001
*L. corniculatus*							
Aboveground biomass	0 (+−0.01)	0.03 (+/−0.01)	−0.25 (+ −0.01)	0.06 (+/−0.01)	0.03 (+/−0.01)	F_4,20_=6.40	0.002
Belowground biomass	0 (+/−0.03)	−0.47 (+/−0.03)	−0.74 (+/−0.03)	−1.02 (+/−0.02)	−0.21 (+/−0.03)	F_4,20_ = 6,75	0.001
Total biomass	0 (+ −0.02)	−0.13 (+/−0.02)	−0.41 (+/−0.01)	−0.26 (+/−0.02)	−0.07 (+/−0.01)	F_4,20_ = 6,39	0.002
