# Supplementary material for: Competition with wind-pollinated plant species alters floral traits of insect-pollinated plant species
Source: Sci Rep. 2015 Sep 3;5:13345. doi: 10.1038/srep13345 (PMC4558602; doi:10.1038/srep13345)
Supplement: Supplementary Information [file srep13345-s1.doc]

**Supplementary information**

Title: Competition with wind-pollinated plant species alters floral traits of insect-pollinated plant species.

Floriane Flacher, Xavier Raynaud, Amandine Hansart, Eric Motard and Isabelle Dajoz

Supplementary Figure S1: Mean (+/-standard error) biomass (g.plant^-1^) of: *E. plantagineum, L. purpureum* and *L. corniculatus* in mixture with the competitors (three top panels), and competitor species in mixtures with *E. plantagineum*, *L. purpureum a*nd *L. corniculatus* (three bottom panels). Above-ground biomass (light grey, P *_L. purpureum_,* and *P_E. plantagineum_* <0.01, P *_L. corniculatus_* =0.06; P_competitors with_ *_L. purpureum_*, P_competitors with_ *_E. plantagineum_*, P_competitors with_ *_L. corniculatus_* <0.01), below-ground biomass (dark grey, P<0.01 for all) (ANOVA, N=75 for each test). “Mono” refers to monocultures of the focal species. Wind-pollinated species are ordered according to increasing intensity of competitive interactions (see Results). Different letters are for significant differences (Tukey all pair comparison test and adjustment of p-values, Holm method^56^) (bold letters: total biomass; letters at the top: above-ground biomass; letters at the bottom: below-ground biomass).

Supplementary Figure S2: Linear regression between the total flower production and mean ln RR values per plant calculated from final biomass (P<0.001 with transformed data for all three species; *E. plantagineum* R²=0.52, *L. purpureum* R²=0.70 and *Lotus corniculatus* R²=0.21). The grey line refers to the model after data transformation while dots represent untransformed data. Ln RR values are associated to mixtures in the legend.

Supplementary Figure S3: Mean (+/- standard error) flower size (mm) of *E. plantagineum, L. purpureum and L. corniculatus* in mixture with the competitors. “Mono” refers to monocultures of the focal species. Wind-pollinated species are ordered according to increasing intensity of competitive interactions (see Results). *E plantagineum:* N= 806, F_4,772_= 7.35, P<0.001; *L purpureum*: N= 1971, F_1,1936_= 8.96, P<0.001; *L corniculatus:* N= 1070, F_4,1036_ = 4.69, P<0.001 (ANCOVA). Different letters are for significant differences (Tukey all pair comparison test and adjustment of p-values, Holm method^56^)

Supplementary Figure S1:

Supplementary Figure S2:


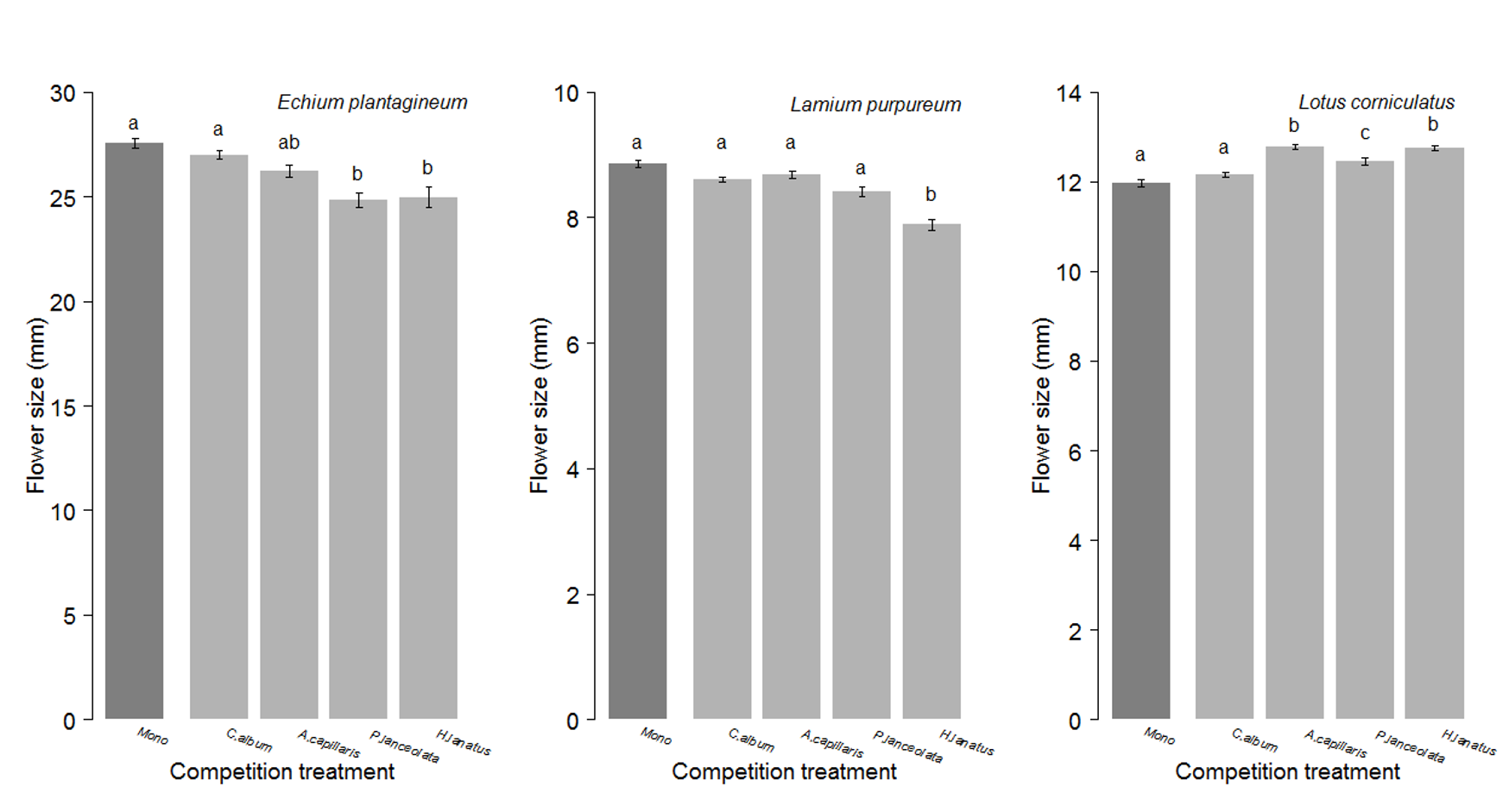
Supplementary Figure S3:

Supplementary Table S1: Plant species description ^1-3^ (Family names are given according to APG III classification^4^)

| Plant species | Status | Life cycle | Habitat | Habitat preference based on Ellenberg values^5^ | Other characteristics |
| --- | --- | --- | --- | --- | --- |
| Insect-pollinated species |  |  |  |  |  |
| *Echium plantagineum* L.  (Boraginaceae) | native to the western Mediterranean basin subspontanous in the region | annual or biennial | along coasts, sandy places | full light  intermediate fertility intermediate pH | violet tubular flowers mainly pollinated by honeybees, bumblebees, butterflies |
| *Lamium purpureum* L.  (Lamiaceae) | native to the region | annual | grasslands, forest hedges | intermediate light  high fertility  intermediate pH | pink tubular flowers mainly pollinated by honeybees, bumblebees |
| *Lotus corniculatus* L.  (Fabaceae) | native to the region | perennial | grasslands | intermediate to full light  low fertility  low to intermediate pH | nitrogen fixer;  yellow tubular flowers mainly pollinated by bumblebees |
| *Mimulus guttatus* DC.  syn. *Erythranthe guttata* (Fisch. ex DC.) G.L.Nesom  (Phrymaceae) | Subspontaneous in the region | perennial | grasslands, streambanks | intermediate to full light  intermediate fertility  intermediate pH | yellow tubular flowers mainly pollinated by bumblebees and solitary bees |
| Wind-pollinated species |  |  |  |  |  |
| *Agrostis capillaris* L.  (Poaceae) | native to the region | perennial | grasslands,  wastelands | intermediate light  intermediate fertility  low to intermediate pH |  |
| *Chenopodium album* L.  (Amaranthaceae) | native to the region | annual | grasslands,  wastelands | intermediate to full light  high fertility  intermediate pH |  |
| *Holcus lanatus* L.  (Poaceae) | native to the region | perennial | grasslands,  wastelands | intermediate to full light  intermediate fertility intermediate pH |  |
| *Plantago lanceolata* L.  (Plantaginaceae) | native to the region | perennial | grasslands,  wastelands | intermediate light  intermediate fertility intermediate pH |  |

**REFERENCES of Supplementary information**

1. Coste, H. "Flore de la France." *Paris: Librairie des Sciences Naturelles* (1901).

2. Hanley, M. E. & Goulson, D. Introduced weeds pollinated by introduced bees : Cause or effect ? *Weed Biol. Manag.* **3,** 204–212 (2003).

3. Free, J. The flower constancy of bumblebees. *J. Anim. Ecol.* **39,** 395–402 (1970).

4. Chase, M. W., Reveal, J. L., Hortorium, L. H. B., Biology, P. & Building, M. A phylogenetic classification of the land plants to accompany APG III. *Bot. J. Liennean Soc.* **161,** 122–127 (2009).

5. Hill, M., Mountford, J., Roy, D. & Bunce, R. *Ellenberg’s indicator values for British plants. ECOFACT Volume 2 Technical Annex*. (1999).
